# Towards an Evolutionary Model of Animal-Associated Microbiomes

**DOI:** 10.3390/e13030570

**Published:** 2011-02-25

**Authors:** Carl J. Yeoman, Nicholas Chia, Suleyman Yildirim, Margret E. Berg Miller, Angela Kent, Rebecca Stumpf, Steven R. Leigh, Karen E. Nelson, Bryan A. White, Brenda A. Wilson

**Affiliations:** 1 Institute of Genomic Biology, University of Illinois, Urbana, IL 61801, USA; 2 Department of Physics, University of Illinois, Urbana, IL 61801, USA; 3 Department of Natural Resources and Environmental Sciences, University of Illinois, Urbana, IL 61801, USA; 4 Department of Anthropology, University of Illinois, Urbana, IL 61801, USA; 5 J. Craig Venter Institute, Rockville, MD 20850, USA; 6 Department of Animal Sciences, University of Illinois, IL 61801, USA; 7 Department of Microbiology, University of Illinois, IL 61801, USA

**Keywords:** microbiome, evolution, animal, multi-level selection, modularity, complexity, interdependency, ecology

## Abstract

Second-generation sequencing technologies have granted us greater access to the diversity and genetics of microbial communities that naturally reside endo- and ecto-symbiotically with animal hosts. Substantial research has emerged describing the diversity and broader trends that exist within and between host species and their associated microbial ecosystems, yet the application of these data to our evolutionary understanding of microbiomes appears fragmented. For the most part biological perspectives are based on limited observations of oversimplified communities, while mathematical and/or computational modeling of these concepts often lack biological precedence. In recognition of this disconnect, both fields have attempted to incorporate ecological theories, although their applicability is currently a subject of debate because most ecological theories were developed based on observations of macro-organisms and their ecosystems. For the purposes of this review, we attempt to transcend the biological, ecological and computational realms, drawing on extensive literature, to forge a useful framework that can, at a minimum be built upon, but ideally will shape the hypotheses of each field as they move forward. In evaluating the top-down selection pressures that are exerted on a microbiome we find cause to warrant reconsideration of the much-maligned theory of multi-level selection and reason that complexity must be underscored by modularity.

## 1. Introduction

Scientific theory on the origin of life remains obscure, with several fundamental aspects still lacking a clear resolution. It remains to be determined whether life evolved under extreme volcanic or hydrothermal conditions or conversely under much colder conditions [1,2]; whether the earliest life forms were heterotrophic or autotrophic [3]; whether replication preceded metabolism; or whether cells were an early or late part of the equation [4]. Nevertheless, life evolved and became prolific. Microbes, a term used here to encompass members of both the bacterial and archaeal lineages (as per [5]), are almost certainly more complex than the earliest forms of life, yet microbes constitute the earliest branching life forms of all extant organisms. Early microbes had advanced sufficiently as to form spatially organized communities capable of leaving distinguishable fossils up to 3.43 billion years ago [6]. Extant microbial populations collectively are estimated to number at least 4,000,000,000,000,000,000,000,000,000,000 (4 quintillion or 4 × 10^30^) cells [7], making them the most abundant life form on Earth. The current fossil records suggest that the dawn of Animalia occurred much more recently during the Ediacaran period, an epoch occurring 635–541 million years ago (Mya) [8,9]. Bilaterian animals subsequently flourished and diversified during the Cambrian (542–488 Mya) in an event referred to as the Cambrian Explosion. Our primordial animal relatives may have had little to fear in terms of microbial pathogenesis, which would have taken some time to evolve. Yet, early development of the kingdom Animalia would have taken place in what has been called a ‘microbial soup’ [10], where both pathogenic and mutualistic relationships would have ultimately evolved. The subsequent evolution of processes enabling the selective retention of certain colonizing microbes, principally those that conferred a benefit, or limited the colonizing ability of those with pathogenic tendencies or both would have dramatically increased host fitness and would likely have been favored by strong selective pressures. It has been suggested that those microbes that have been selectively maintained have then co-evolved with their animal hosts, and this has led to specialization and an increased reliance of the host and its microbiome upon each other. Numerous examples of host-microbe co-evolution have been described, with the most stringent being the obligate mutualisms that occur in several insects including the associations of *Wigglesworthia glossinidia* with the tsetse fly [11], *Buchnera aphidicola* with aphids [12], and *Blochmannia pennsylvanicus* with ants [13]. In these obligate mutualisms, the bacterial species cannot live without their hosts and the viability and reproductive success of their hosts are highly dependent on their endosymbiotic bacteria [14].

An animal host “ecosystem” possesses numerous epithelial niches that are (or will be) colonized by a site-specific microbiome comprised mostly of bacteria. Dependent on the location of the niche and the specific animal host, microbiome colonists may also include archaea, fungi and protozoa. Bacteriophages are constituent members of all microbiomes, where they are thought to make substantial contributions to the maintenance of community structure and to the evolutionary trajectories of the inhabitant microbiome [15].

The ecological relationships that have evolved between the animal host and its microbiome are, for the most part mutually beneficial. For the microbiome, the animal provides a typically eutrophic and relatively stable environment (at least in terms of pH, redox potential, water activity and for internal sites temperature), while a desired microbiome positively influences host health. A clear example of this is observed in the gastrointestinal (GI) tracts of mammals. The principal benefit afforded by the GI microbiome is the ability to harvest otherwise inaccessible nutrients from recalcitrant fibers in foods, converting these substrates to energy rich short-chain fatty acids (SCFA) that are then absorbed and used by the host [16]. One of these SCFAs, butyrate, is itself attributed numerous health promoting properties including the physiological development of the GI tract [17]. Overall microbially produced SCFAs are estimated to provide up to 70% of an animal’s daily energy intake [18]. The overall production of SCFAs has been shown to correlate with both alterations in the taxonomic structure of the GI microbiome and human obesity [19]. In both host-associated and in natural ecosystems, microbial processes are responsible for the maintenance of ecosystem health. The mammalian GI microbiome has been shown to make additional contributions to host health by producing vitamins [20], metabolizing, and modulating host responses to xenobiotics [21,22], attenuating inflammatory responses [23], increasing resistance to pathogenic bacteria, assisting in the development of the immune system [24], development and function of the brain [25], and modulating behavior [25–27]. Although less well described, microbiomes found in other animal-associated niches are equally intriguing in their health-promoting properties. The vaginal microbiomes of healthy humans, for instance, tend to be dominated by lactic acid-producing *Lactobacilli*. The activity of the *Lactobacilli*, at a minimum lowers the pH to <4.5, a condition that is unfavorable for many potential vaginal pathogens. Disturbances in the vaginal ecosystem that reduce the dominant *Lactobacilli* can predispose the host to pre-term birth and bacterial vaginosis; a condition that itself correlates with infertility and an increased risk of sexually transmitted infections [28–30]. The naive human vagina, sterile at birth, therefore depends upon the *Lactobacilli* for maintaining health. This, in itself, is indicative of the mutualistic relationship that has co-evolved between host and microbiome. Perturbations of the typical taxonomic structure of a microbiome (such as through pathogenic invasion and/or opportunistic pathogens) can alter its ecological function leading to numerous negative influences on health. Causative links have been made between perturbed microbiomes and numerous diseases and disorders that include, but are not limited to: peptic ulcers [31], kidney stones [32], neurological disorders (including memory dysfunction, Tourette’s syndrome and obsessive compulsive disorder) [26,33,34], cancer [31], osteoporosis, cardiovascular disease [35], obesity [19,36], pre-term birth and diabetes [35,37]. Consequently, much investment is being made into understanding the constituents and functional contributions of, in-particular human-associated microbiomes [38], but also our direct evolutionary relatives and other members of the kingdom Animalia.

Preliminary sequencing-based studies on microbiome composition and/or metabolic potential (by virtue of genetic content) have been made on the GI including oral, illeal, colonal, distal gut and fecal (used as a proxy for the post-pharyngeal portion of the GI tract, although probably only providing a useful sample of the diversity from the colon onward) microbiomes, as well as the dermal, vaginal, penile, ear and/or eye microbiomes of mammals, including humans [39,40], other primates [41,42], Diprotodonts [43]; Artiodactyls [44] and members of the order Carnivora [45]; Reptiles [46]; Aves, including Struthioniformes [47] and Galliformes [48]; and Insects of the order Isoptera [49] and Hymenoptera [50]. Alongside, countless microbial reference genomes have been, and are continuing to be sequenced [51]. Consequently, a large amount of biological information is becoming available that may ultimately be used to test the evolutionary dynamics of microbiomes. To date our comprehension of microbiome evolution has been drawn largely from the studies of artificially produced ecosystems that do not replicate *in vivo* complexity; digital organisms that are confined to user-defined parameters, which are based on our limited knowledge; and ecological theories designed for larger sexually-reproducing macro-organisms. Theory provides an essential framework for developing and testing our understanding of community assembly and the interactions among microbial populations and their environment. Ecological theories that have been developed to generalize observations of the natural history of plants and animals may poorly accommodate the diversity, abundance, short generation time, and keystone ecological functions of microbial communities [52]. In addition, such theories do not account for the extensive Horizontal Gene Transfer (HGT) that is a hallmark of microbial communities and a primary driver of both microbial ecology and evolution. The difficulties in observing microbial communities in their natural settings and distinguishing populations have contributed to the disconnect between microbiology and ecological theory [52]. In spite of their limitations, each of these fields has contributed substantially to our understanding of microbial evolution. By carefully drawing on each we aim to produce a more unified theorem of the ecological evolution of microbiomes to enable the formation of hypotheses for subsequent experimentation. Hypothesis directed experimentation should ultimately lead to a more comprehensive and accurate model describing microbiome evolution. At an applied level a fuller understanding will have pronounced consequences for applied fields, such as medicine and ecology where scientific advancement is thought to have been retarded by a lack of evolutionary appreciation [53].

## 2. Evolutionary Pressures within a Microbiome: A Background

Animal-associated microbiomes are non-equilibrium dynamic systems. Understanding their evolutionary trajectories requires consideration of the selective and other evolutionary forces and the different scales at which they apply to the microbes that occupy the microbiome. These evolutionary forces are numerous, can act across multiple scales that appear strongly coupled, and be variable over space and time. Based on the available evidence we have classified the evolutionary forces applied to animal-associated microbiomes into three broad categories that we term the primary, secondary and tertiary evolutionary pressures.

### 2.1. Intraspecific Competition—The Primary Evolutionary Pressures

We define the primary evolutionary pressures as those that occur intraspecifically, driving evolutionary trajectories of each microbial species. A microbial species is not clonal, but rather a species is represented by a broad spectrum of genetic variants that radiate from a central clonal-genotype. Because of the asexual nature of microbes and their consequential equal propensity to reproduce, the central clonal genotype should comprise the modal subpopulation. Variants continually arise through mutation, which occur at approximately 10^−7^/bp/replication [54]. This rate can be higher when selection pressures are continuously changing [54] or in the presence of mutagens. In addition, microbes are amenable to HGT, which appears to be of considerable influence in intraspecies genomic variation as is evidenced by the numerous reference genomes generated for single species that display not just allelic differences, but differences in whole sets of genes [55]. In fact, analyses early in the genomics era found evidence of HGT affecting between 1.5 and 14.5% of all genes in complete microbial genomes [56]. More recently it has been shown to affect more than a third (~34%) of all gene families [57]. Species within microbiomes do not escape this trend, with several studies providing evidence that HGT is pervasive within at least the GI [58] and vaginal [59] microbial ecosystems. Intraspecifically, HGT can lead to rapid evolution of novel functionality [60] and drive important ecological events such as speciation [61] and modularity [62].

Population genetics theories incorporate the assumption that periodic bouts of strong selection (“sweeps”) act to purge less ‘fit’ variants, essentially suggesting that a species goes through cycles of broader intraspecies variation followed by periods of clonality. However, in evaluating the literature we, and others [63], find little evidence to support this phenomenon outside of pathogenic microbes [64]. This lack of evidence may stem from the relative scarcity of research into intraspecific dynamics of non-pathogenic organisms, however it should also be noted that pathogens, unlike non-pathogenic microbes, are subject to a unique evolutionary arms race with the host. In defining the evolution of a microbial species, we specifically refer to the tendency of each genetically homogenous (or clonal) subpopulation of a microbial species (each variant) to shift the mode in subsequent generations. Within a species, this is largely driven by growth rate, as variant subpopulations with faster growth rates will over generational time command a greater share of the species population, tending toward the mode. Within the microbial realm, growth rate is directly influenced by the amount of excess energy available to the microbe after satisfying its metabolic and biosynthetic requirements (maintenance energy), as has been demonstrated experimentally [65–67]. That is to say growth rate is influenced by the availability and quality of catabolites, the efficacy of the microbes in obtaining and processing the catabolites, minus the costs associated with obtaining and processing the catabolites into energetic currency (*i.e.*, ATP), as well as the organisms auxiliary anabolic and biosynthetic requirements. Historic measurements indicate that microbial biomass increases in the range of 6.3–19 g cell protein/mol ATP generated, depending on maintenance requirements [68]. In addition, the efficiency with which these processes occur (as well as genome replication and cell division) is also of considerable influence. For example, where the rate of substrate acquisition (nutrient harvest rate) exceeds the rate with which it is processed and converted into biomass (conversion efficiency), energy that would otherwise be available for growth (and work) is lost as heat [69]. Although such energy dissipation may be of value in limiting the growth potential of other species exploiting the same resource (competitors), it also reduces the maximum biomass potentiated by the more ‘inefficient’ species. Thus variants with mutations that positively affect any aspect of growth rate, including the efficiency of catabolism, have the potential to shift the mode. While beyond the intended scope of this review, it should be noted that fewer beneficial mutations exist than those that are neutral or that reduce fitness (see Extreme Value theory; [70,71]) and that adaptation favors mutations conferring larger shifts in fitness due to the processes of genetic drift and clonal interference [72–75]. It is therefore noteworthy, that the process of HGT, which can facilitate the transfer of whole genes or even sets of genes, is in most instances likely to cause greater shifts in fitness than point mutations [60]. This is because the transfer of whole genes results in more meaningful differences in phenotype. Despite the potential for larger mutations, such as those conferred by HGT, for enhancing genomic entropy, selection on the gene level instead enhances long-range order in the form of repeated domains and operons [76]. Since, in this sense, HGT shuffles ordered elements, it facilitates greater rates of microbial adaptation.

### 2.2. Ecological Interactions—The Secondary Evolutionary Pressures

We define the secondary evolutionary pressures as those driven by the ecological interactions among the community members of the microbiome [77]. These interactions include interspecific competition and predation, both of which have long been recognized as important selective features driving evolutionary trajectories, but also symbiotic interactions which are only more recently beginning to be fully explored for their contributions to evolution, speciation and adaptation [78]. Symbioses between two species may be beneficial to one (commensalism) or both (mutualism) with no negative effect on either, or conversely be advantageous to one, while of considerably detrimental to the other (parasitism).

Interspecific competition for resources plays a substantial role in driving evolutionary trajectories, and has been proposed to drive ecological structure [78]. When competition exists for growth-limiting resources, particularly those of the lowest abundance (the law of the minimum [79]), the succession of a species within the ecosystem is dependent on the competitive ability of each population for available niches. This definition recognizes the role of the environment in shaping both ecological structure and evolutionary trajectories. Competition can manifest in several evolutionary adaptations; as with the previously described primary selective pressures, faster growth rates can afford a species a larger share of the resources. However, due to the larger genetic differences between species that can result in more substantive phenotypic differences in resistance mechanisms and/or susceptibilities, invoking additional biological interactions, such as amensalism can be used to circumvent advantages in the rates of growth and/or nutrient harvest. The production of toxic compounds, such as bacteriocins is an example of this strategy [80].

**Figure 1.**
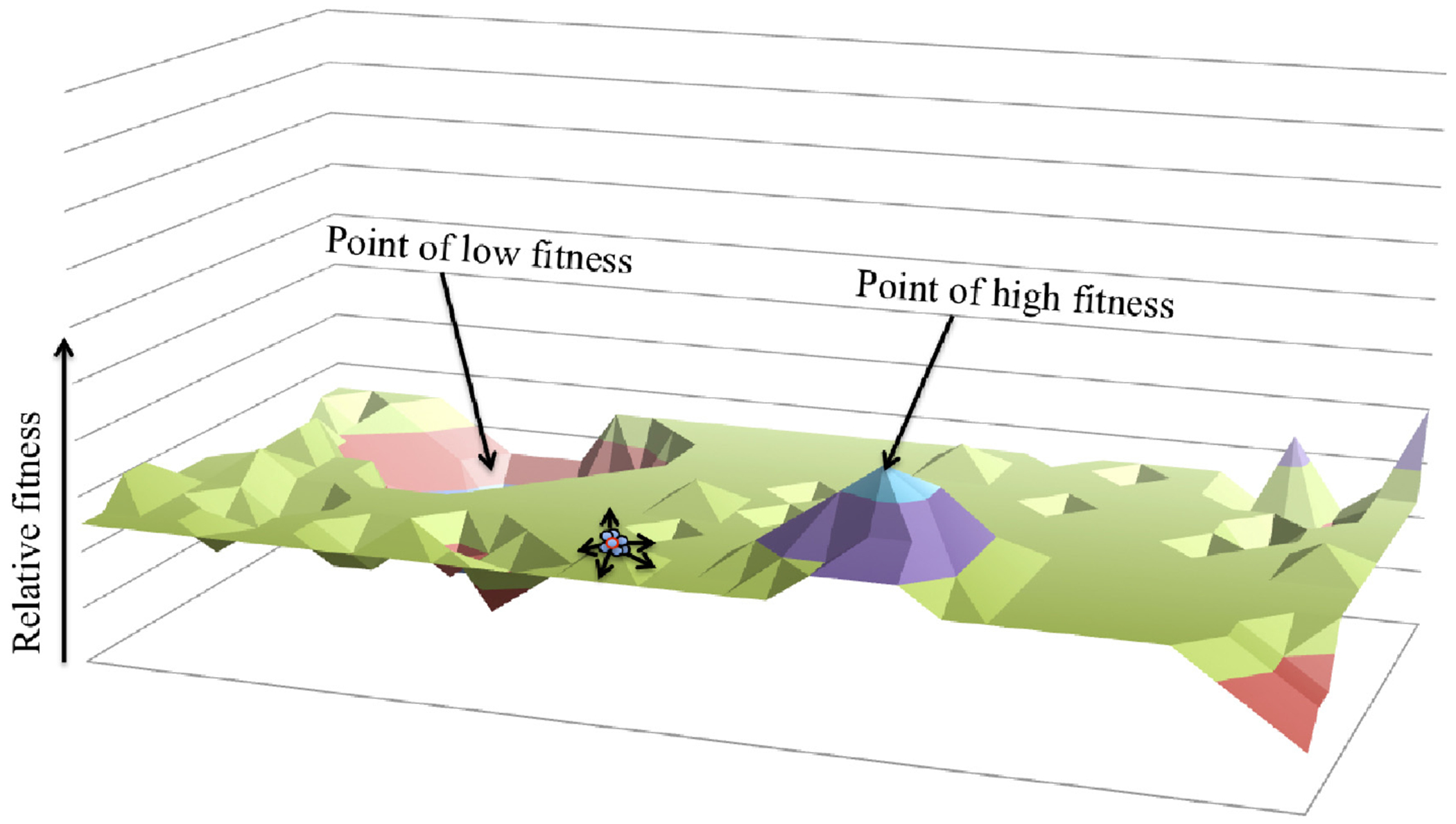
Fitness Landscape. A 3-dimensional representation of a fitness landscape; used to describe the multiplicity of adaptive trajectories available to a species. These trajectories may lead to points of lower fitness, represented as a valley, or higher points of fitness, represented as a mountain, while neutral regions manifest as plateaus. While a clone would occupy a single point within the fitness landscape, a species is aclonal. Although best thought of in an additional dimension, for simplicity we have depicted a species (blue dots) as multiple clonal types surrounding the modal subpopulation (circled in red). Adaptation arises when the modal subpopulation moves to a new position within the landscape although intraspecific genetic variation would enable tethered migrations from the modal genotype.

Competition may drive specialization, in the sense that all niches are subject to interspecific competition, and an organism that attempts to succeed in multiple niches (a generalist) has more directions to explore in a fitness landscape (a theoretical means of envisaging adaptive potential, where peaks represent points of higher fitness and valleys represent lower fitness [81]; see Figure 1) than one evolving to exploit a single niche (a specialist). Competition may have also driven species to exploit less rewarding niches. After accounting for amensalistic effects, competitive exclusion theory would suggest that species with the fastest growth rates ultimately displace slower growing species within a niche [82,83]. Therefore a species that is unable to compete within a niche of high nutritive value may mitigate or modify competitive influences by instead occupying a separate, less nutritive niche of limited overlap.

The contribution of competition to community structure has, however, undergone considerable debate and led to the spawning of the neutral theory of biodiversity, which implies that all niches are equivalent and the final composition is purely shaped by random immigration events, cell division (birth) and death [84].

The neutral theory of biodiversity works surprisingly well with macrobial ecosystems and with some basic modifications has recently been found to have reasonable predictive power for free-living microbial ecosystems [85]. Yet, the neutral theory of biodiversity does not predict community composition for the fecal microbiome [85,86]. We expect the same is true of other animal-associated endosymbiotic microbiomes based on the following findings:

1. The primary colonizers of mammalian hosts, which are acquired from the environment (principally from the mother), are rapidly replaced over the first year of life [87];
2. Different body sites host distinct and predictable microbial assemblages [88].

These observations suggest that niche theory may be a better model for host-associated microbiomes. Mutualistic and commensal relationships among microbes largely manifest as interdependencies. A large proportion of microbes within an ecosystem are known to exist in a state of metabolic interdependence [89–92]. Over evolutionary time, members who consistently co-localize within a microbiome may dispense with the enzymatic machinery required for the production, acquisition or enrichment of certain key metabolites, such as vitamins, minerals, amino acids, depolymerized sugars or the various cofactors, and instead rely on the other member(s) who overproduce (or enrich) the metabolite as a by-product or end-product (subsequently grouped as ‘by-products’) of their own metabolism. The dependent member may then reduce its metabolic cost by simply electing to harvest these by-products from the environment, or potentially more directly through co-association. Such cross-feeding relationships have been shown to occur experimentally [90–93]. Interdependencies have been theorized to evolve such that metabolic pathways are optimized, maximizing the rate of ATP production, while minimizing the total concentration of enzymes required and intermediates produced [94]. That is to say, the generation of ATP, or other reducing equivalents, and the associated costs are not uniform across an entire metabolic pathway. If an organism can participate in just the most beneficial region of a pathway (which may occur at the beginning, the end or anywhere in-between) passing on or forgoing the other less desirable portions of the pathway, this would be of substantial benefit. The advantages of niche specialization (discussed above) would, for some species, promote evolutionary investment in less profitable regions of a pathway. In addition, the most beneficial region of a pathway is not always the same for each organism; for instance an organism that utilizes two metabolic pathways with overlapping metabolic intermediates may be less efficient in one or both pathways than an organism that utilizes just one of the pathways due to the variable efficiency of the enzymatic machinery involved in the overlapping portions of the pathways. This line of reasoning might lead to the expectation that microbial community diversity and metabolic efficiency would be positively correlated at the community level.

Interdependencies also enable global compartmentalization of metabolic pathways, a central paradigm in flux balance analysis [95]. Further still, interdependencies would be favored when the energy expenditure associated with maintaining and operating the enzymatic machinery necessary to biosynthesize a metabolite exceeds that of transporting the same metabolite across the membrane, as is expected for nucleosides [96], provided the metabolite is not limiting in supply (The law of the minimum [79]). This reduction in cost results in an overall improvement in energy available to the microbe, which should have direct consequences on the rate of biomass formation (as discussed above).

Metabolic interdependencies may additionally contribute in a self-perpetuating manner to the ecological structure of a microbiome through niche construction, a factor that if subject to selection (as implied by multi-level selection; described below) would negate the neutral theory of biodiversity. Microbial metabolisms that make available by-products that were previously unavailable or limited within the ecosystem may be exploited to influence both the local and global structure of the microbiome, as these new metabolites help to create new (niche creation), or modify existing (niche modification) niches, thereby making the ecosystem accessible (or conversely inaccessible) to a new subsection of microbes, as has been demonstrated experimentally [97] and is known to occur *in vivo*.

An interesting example of niche modification occurs in the human vagina, where the production of lactic acid by naturally colonizing *Lactobacilli* reduces the pH and reduces the colonizing ability of other microbes that are principally considered as pathogenic [98], resulting in a system that is dominated (70–90%) by *Lactobacilli spp*. [39]. An example of niche creation is observed in the GI tract with ‘hydrogen sinks’, including methanogenic, sulfate-reducing (SRB) and/or acetogenic microbes. These microbes subsist on the hydrogen and, with the exception of SRBs, CO_2_ produced as common by-products of carbohydrate metabolism by anaerobic microbes [16,99]. This later example shows that a freshly created niche can be exploited by one of three different metabolisms, each resulting in the production of distinct by-products. Each of these distinct by-products can thereby produce, or further modify, other niches. Theoretical studies have found niche construction can significantly affect evolutionary and ecological dynamics [100]. Through scaling of the evolutionary properties of niche construction applied to single species, niche construction can be expected to promote ecological inertia, reduce ecological entropy, favor interrelationships that would otherwise be unfavorable and eliminate interrelationships that would otherwise be favorable [100].

As many animals are born or emerge sterile and must selectively acquire their microbial colonists from the environment, early colonists potentially have considerable influence over the subsequent development of the ecosystems (through creation/modification of new niches) and succession of microbial populations. It would then follow that as succession within a particular microbiome proceeds, the probability that a new colonist is dependent on the metabolisms of the preceding microbiome increases, as does the likely extent of their co-dependency. Ultimately, assembly of the microbiome should reach a sub-terminal point (a ‘sink’) where the available metabolites are no longer of sufficient nutritional value to maintain a positive growth rate. The specific microbes occupying this sub-terminal point are expected to be more variable than other niches over time, being influenced by the rate of immigration of and/or adaptation by rapidly growing indigenous members of the microbiome, to metabolically-capable microbes (the ‘source’, as per the theory of source-sink dynamics [101]). The resulting taxonomic structure of the microbiome is one intertwined with ‘trophic webs’ through which carbon, nitrogen, and other key metabolites flow [102]. Where mutual benefit is derived from nutritional interdependency, co-evolution may act to promote specificity among metabolic partners that are self-stabilizing.

As a collective consequence the ultimate number of niches within a microbiome should be strongly influenced by the metabolisms of the inhabiting microbiome. By virtue of the vast array of microbial metabolisms and the expanse of the mobile gene pool available in nature, all niches of sustainable nutritive value that can be filled will almost certainly be filled, and those that are not readily filled provide an opportunity for evolutionary adaptation by members of the resident microbiome, as has been observed in controlled experiments [91], particularly those exploiting the most lucrative niches [101]. Consequently, irrespective of the dynamism of a microbiome, only a finite number of taxonomic structures should theoretically exist, these being most dependent on the primary colonizers and proportionately less by subsequent colonizers. Observation of this phenomenon might be masked by functional redundancy, or the interspecific overlap of niches (defined in the Eltonian sense) [103,104].

Endosymbiotic microbiomes of all animals tend to be dominated by Firmicutes, Bacteroidetes, and Actinobacteria. Despite the conservation of these microbial phyla across animalia, current surveys have suggested less commonality at the strain and species levels where cohesion should operate [105]. Over smaller phylogenetic distances, genetic heterogeneity is more prevalent within the fecal microbiome than is observed over the same distances in free-living microbes [106] This suggests that short-term evolutionary adaptation of the resident microbiome is pervasive and plays, at a minimum, an equally important role as the acquisition of niche-filling microbes from the regional species pool. Phenomena such as dramatic increases in transposon density in bacteria following host-restriction [107], which is posited as a means to enable rapid and substantial evolutionary adaptation via gene duplication [108], are observed following host restriction, suggesting that the potential for colonization of a microbiome is remarkably constrained.

HGT is presumed to be an important driver of rapid short-term adaptation of the microbial residents of microbiomes. Given the apparent pervasiveness of HGT in these ecosystems, one of the most controversial aspects of the neutral theory of biodiversity becomes plausible. Based on the findings of Zaneveld [106], a convincing argument can be made that HGT facilitates niche-equivalency. An important caveat is that this argument is based on the assumptions that all species have an equal propensity to acquire the necessary functions from the horizontal gene pool and can then utilize those functions equivalently. It has been proposed that irrespective of the final taxonomic configuration of a microbiome, biogeochemically it will obtain the same entropic state given the biophysiochemical constraints [109]. At the genetic and enzymatic levels, this is also consistent with the notion of functional convergence in spite of species variation [106].

An important consideration of the final taxonomic structure is that not all niches are created equal. As an example, the depolymerization of cellulose and the hemicelluloses inherent in plant biomass within the GI tract represents an energetically expensive process requiring the synthesis and co-ordination of substantial enzymatic machinery [110]. Accounting for these costs and rates of hydrolysis, compared to the catabolism of the more simple sugar substrates, the deconstruction of these polymeric carbohydrates results in proportionately less energy profit per unit of time. Therefore, particular members of the microbial assemblage grow much faster than others, although clearly this is not proportionate to their importance to the function and capacity of the ecosystem, and equally fast-growing populations do not necessarily represent “keystone species”. Competitive exclusion theory would suggest that organism(s) with the fastest growth rates would ultimately displace slower growing members with overlapping metabolic requirements [82,83], noting that metabolic requirements extend beyond the energy source of a microbe. Variability in available energy sources or exclusion of the aforementioned keystone species would alter the niche and lead to succession in the microbial community. Fast-growing community members may also be especially vulnerable to predation.

The major microbial predation within microbiomes is the result of bacteriophage, which numerically out-number microbes by an order of magnitude in most ecosystems [63]. In addition to phage, protozoa are also observed in some animal-associated microbial ecosystems [17,111]. Phage predation, in particular, is considered to be a critical factor preserving microbial diversity. Predation by individual phage populations is species-specific; consequently, more abundant or actively growing microbes draw an unfavorable skew in the viral load, leading to a situation often referred to as the ‘killing the winner’ concept. This concept, supported by *in-vitro* simulations [112] along with *in vivo* observations [113], affords microbes protection to adopt distinct lifestyle strategies and is important in uncoupling competition between overlapping niches. However, phage predation is also predicted to have an oscillatory effect on microbial densities due to the predator:prey (phage:bacteria) arms race that occurs [114]. Bacteria, in a Lamarckian fashion [115], are able to acquire immunity from phage predation, through integrating small segments of the phage nucleic acid into their specific viral libraries known as Clustered Regularly Interspaced Palindromic Repeats (CRISPRs). CRISPR-associated elements (Cas) proteins can then use the CRISPR library to recognize and inactivate the phage on subsequent infection [116]. On the other side of the battle, phage, through necessity for their continued persistence, must then adapt to avoid recognition. Unlike phage predation, protozoan predation is not generally considered to be species specific, though some taxa are preferentially consumed in free-living systems, possibly due to size constraints [117] as bacterivory by protists is size dependent. In addition, actively growing populations appear to be more susceptible to protozoan predation [118]. The major influence of protozoan predation on microbial evolution is driving the microbiome towards a biofilm lifestyle [119] or alternatively a larger cell size [120]. Merely based on probability, it is expected that predation pressures would be favorable to less populous and slower-growing microbes.

### 2.3. Host Influences–The Tertiary Evolutionary Pressures

The tertiary evolutionary factors we define as those factors applied by the host. Such effects are supported by numerous studies that have shown different animal species, even those that are closely related, possess host-specific microbiomes [41,42,83,121–123] and by the finding that strains of a single microbial species that are isolated from different host species are genetically distinct and more adept for survival in their host of origin [10]. Diet is of critical importance to many, if not all microbiomes. Diet directly influences the type and abundance of the primary metabolites available to the microbiome within the GI [124], and urogenital tracts [125]. Dietary effects are also likely to radiate out to other animal-associated microbial ecosystems, especially as a result of their influence on health and physiology. It should also be pointed out that metabolite distributions within a microbiome are not necessarily homogenous, consequentially spatial and temporal dynamics are known to exist in many microbiomes [39,126,127]. These affect the composition and stability of microbial communities on a finer scale. Analyses of subdivided sites of a microbiome have revealed site-specific enrichment of particular microbes. For ectosymbionts, there appears to be a much greater degree of compositional variation between sub-sites and hosts [88] owing perhaps to a greater environmental exposure and less homogenous conditions.

In addition to diet, other factors clearly exist. Host-mediated selective pressures appear to follow phylogeny, as described in the pioneering work on the GI microbiome of Ley and colleagues [86,123]. More recent work has even suggested phylogeny may exhibit a greater influence than diet [128]. A clear example of this is seen in the Giant and Red Panda fecal microbial ecosystems. Despite the pandas dietary separation from their phylogenetic relatives, who subsist carnivorously, to herbivory, pandas are much more similar to their phylogenetic relatives than to other herbivores [86,123]. The totality of the selective pressure(s) exerted by the host on its microbiome, however, still requires further resolution. Host anatomy undoubtedly has a marked, although less well described effect. For instance, the GI microbiome of foregut or ruminal fermenting hosts show marked distinctions from one another and both vary substantially from hindgut or cecal fermenters [123]. Physiological differences such as transit times and frequency of ingesta in the GI tract affect growth rate optima [129] and population stability [46], respectively. Transit time in particular, may moderate certain microbial metabolisms [130] when considered alongside the rates of substrate availability and growth rate. While anatomy, physiology and immune function (discussed below) are governed by the host genome, which is shaped through phylogenetic descent, many typically unknown host-driven selective pressures are clumped together as host genetics. Correlations have been made between host genetics and the taxonomic structure of the GI microbiome through analyses amongst and between twins [131]. Yet, the inability to distinguish the degree of concordance between monozygotic and dizygotic twins [131] shows the genetic influence is conferred through specific loci rather than the genome as a whole.

In addition to diet, the immune system has received warranted attention. The adaptive immune system is thought to favor endosymbionts that have mutualistically co-evolved with the host [132]. Vaginal epithelial cells secrete IL-6 and IL-8 upon exposure to the vaginal pathogens *Gardnerella vaginalis* or *Atopobium vaginae*, but not to the endosymbiont *Lactobacillus crispatus* [133]. IgA deficiencies in mice have been shown to affect the taxonomic composition of the GI tract microbiome [134]. Toll-like receptors (TLR) are of considerable importance, as they confer the ability of the host to evaluate the taxonomic composition of the microbiome. TLRs have been shown to mediate tolerance to symbiotic microbes and illicit immune responses to pathogens [133]. Studies have linked gene polymorphisms in TLRs to alterations of the taxonomic composition of the GI tract and vaginal microbiomes [135–137]. The selective forces applied by individual members of a single host species also show considerable variation, leading some to question the validity of grouping individuals as replicate ecosystems [85]. Many of the selective pressures are subject to change between individuals of a species, depending on factors such as sex, dominant hand [138], as well as temporally within an individual due to changes in dietary regimes, health status and age [129,139,140].

Importantly, there is considerable potential for evolutionary feedback between microbiomes and their animal hosts. Clearly, the functionality of the microbiome is capable of impacting host fitness as evidenced by their ability to influence health, dietary efficiency (both discussed above) and even mood [141]. The most obvious microbiome for which such influence may have occurred is the GI tract. Diet is implicitly understood to have had a significant effect on evolution and also has a known effect on the community structure of the GI microbiome. Interestingly, potential exists for the microbiome to influence dietary preferences as was revealed by Rezzi and colleagues [142]. Further, the observed effect of the GI microbiome on the efficacy of energy extraction from ingesta suggests that the microbiome could have played fundamental roles in important evolutionary adaptations, such as encephalization. Consider the expensive tissue hypothesis [143]: Improvements in the efficiency of energy extraction from ingesta mediated by shifts in the ecological structure of the GI microbiome could allow for the reduction of the GI tract, a tissue that itself requires substantial energy input. The energy saved could then be diverted to provide power for a growing brain. Microbiomes from other host-associated niches may also exert evolutionary influence over the host. Correlations between the vaginal microbiome and pre-term birth have been reported [28]. Mechanistic links between the vaginal microbiome and gestation period and fetal development are deserving of further investigation.

## 3. Towards an Understanding of Microbiome Evolution

**Figure 2.**
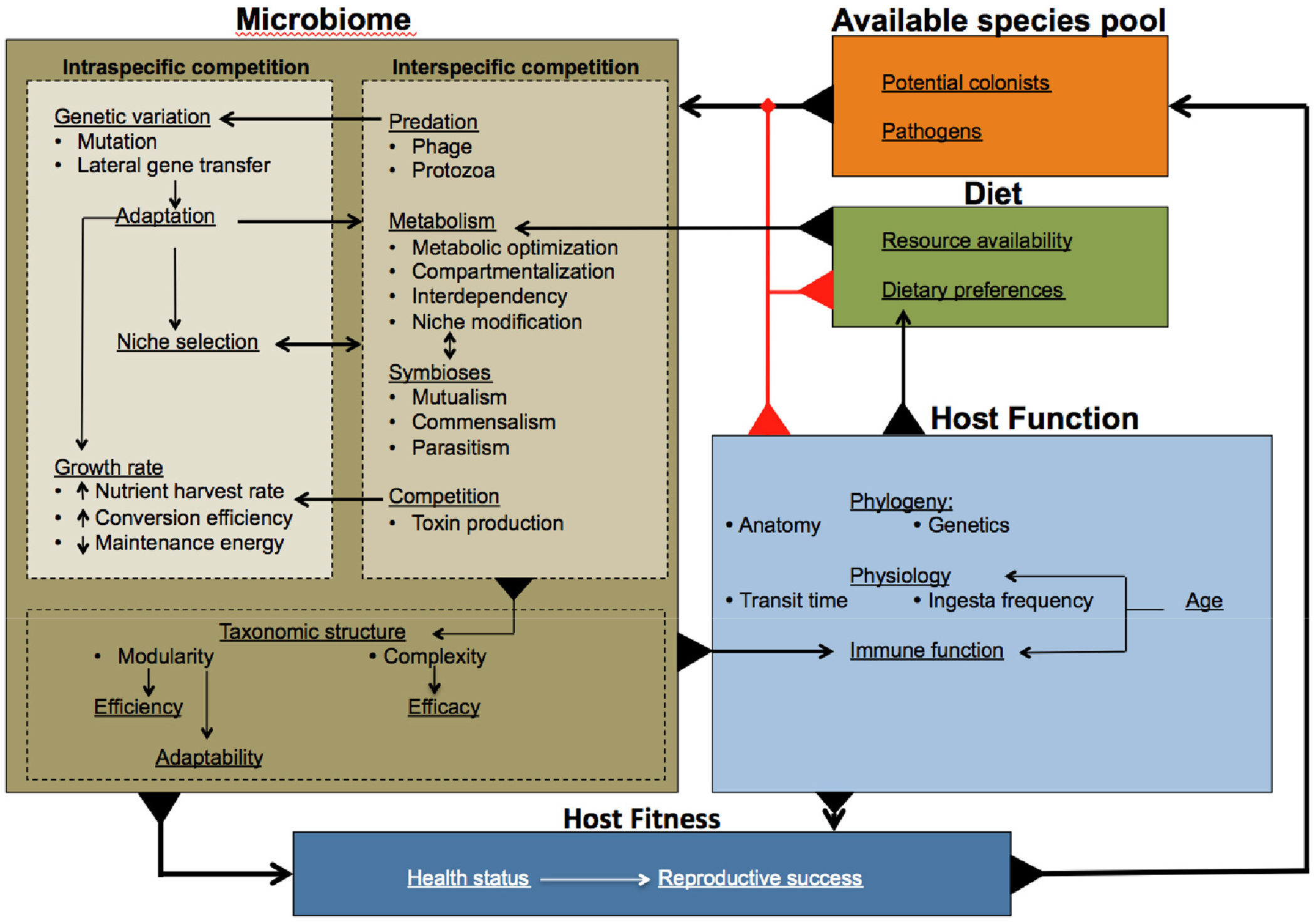
The complexity of selective forces acting on the microbiome. Illustrates the multiplicity of factors influencing the evolutionary trajectories of the microbiome and their interplay as described in this review. Black arrows indicate influence, while red diamonds indicate checkpoints.

The myriad of evolutionary pressures described above already paint a complicated picture of the evolutionary forces that act upon microbial species and community assembly. Yet beyond this, it is widely believed that evolutionary forces also apply at the level of ecosystems [144] with the evolutionary goal, as with all biological systems, to develop a controlled response amidst a background of environmental noise and fluctuation. Replication of biological order on the species level interacts with the disorder introduced by noise and changing selection pressures to produce the appearance of criticality on the ecological scale [145]. To achieve criticality animal-associated microbial ecosystems must work cooperatively and evolutionary forces must act to limit the potential prosperity of ‘cheaters’. Cooperativity may be promoted through ecological complexity but, due to the inherent vulnerabilities (discussed below), complexity must be balanced with modularity. In evaluating the overall picture (Figure 2), we find cause to consider multi-level selection in the evolution of animal-associated microbiomes. Much of the following sections are based on theoretical expectation, due to an absence of research targeting these fundamentally important aspects of microbiome ecology.

## 4. Multi-Level Selection

As our understanding of the complex nature of biological interactions grow, we begin to have greater difficulty discerning clear boundaries between scales. This is especially true for microbial life, where widespread HGT blurs the line between the most exalted of evolutionary divides—the species. The difficulty in defining a firm scale upon which selection acts has in part given rise to a recent resurgence in the theory of Multi-Level Selection (MLS) [146–148]. Although previous applications to macroecology have been much maligned [149], the multiple layers of selection pressures coming from the host, diet, and intra- and inter-specific competition (described above) make the animal microbiome a key system in which to reconsider MLS. MLS, as its name suggests, asserts that selection operates across multiple levels: from the genes, to genomes, to species, to communities. The inability to be competitive at any level will result in elimination, irrespective of how competitive it is at the level below. For microbiomes multiple scales of competition exist, *i.e.*, between genes acquired through HGT or altered through mutation, within and among species, along with the top-down selection pressure from the host. In addition, the host is also subject to natural selection for the most beneficial and functional microbiome. These multiple scales of competition lend themselves to the theory of MLS.

### 4.1. Complexity

Evolution is often described as a march toward increasing complexity [150–152]. At the level of the microbiome, we assume complexity on two levels. Complexity relates to the species richness of an ecosystem, but additionally describes the degree of interdependencies that evolve among members of the ecosystem. As a system becomes increasingly interconnected, it is expected to display increased precision and performance, while becoming less malleable in terms of adaptability [153]. An increase in richness of a microbiome, on the other hand, corresponds to an increased probability that it will be able to fulfill some function. Increases in richness can also lead to functional redundancy of more commonly encoded functions making these parts of the ecosystem less vulnerable [154,155]. Yet paradoxically, when complexity is coupled with an increased interconnectedness it also corresponds to an increase in vulnerability, in the sense that performance of the system is sensitive to the properties of an increased number of specialist (or keystone) functions. The complexity of the system, therefore, has profound consequences for co-evolution, as species must co-evolve in concert with connected species. The problem with this is that a strongly intertwined microbial community structure must move collectively through a fitness landscape, and because it must find a more general solution, the microbiome has more dimensions to explore. Perhaps even more problematic is the expectation that in satisfying the requirements of all niches, an intertwined microbial community would likely find a global optimum that is much less than that potentiated for any particular niche. The only conceivable solution to this is modularity; yet, despite precedence within macrobial ecosystems and their food webs [156] and models that predict it should occur where selection acts within microbes [157], no studies have explicitly explored this in the ecological context of the microbiome.

### 4.2. Modularity

Modularity within a microbiome would manifest as discrete subclusters of interdependent species that would collectively evolve to fulfill a common goal (or set of goals). Modular systems are less vulnerable in that failure of one component does not radiate globally, and they afford the collective system the ability to explore multiple evolutionary landscapes in unison without any cross-hindrance. Modularity has been shown to be a prominent feature of microbes across multiple levels at which selection may act, including gene networks [158], protein-protein interactions [159], and metabolic networks [160].

The main point of contention applied to modularity is how it evolves, through direct natural selection to favor adaptability and/or to limit vulnerability [157] or purely indirectly due to its congruence with other more directly selectable features (‘the congruence principal’) [160]. Modularity seems to be favored by more variable environments [161], which may manifest within the microbiome through pervasive HGT [60] or ecological opportunity [157] akin to that suggested by the study of Zaneveld and colleagues [106]. It seems highly plausible that it would also manifest through a varied diet, of which effects would radiate through the web of interdependency in diet-sensitive niches.

## 5. Cooperation, Altruism and Cheating

The evolution of microbial cooperation is fundamental to realizing the full potential of an ecosystem, yet the potential prosperity of ‘cheaters’ (organisms that reap the rewards produced through the cooperative activity of others without sharing the cost) seeks to constantly undermine this process. Regardless, cooperation [162], and even altruism [163] (although this may conversely be considered cheating by the mass), are often described phenomena within the microbial realm. Mechanistically, the theory of kin selection (the costs of cooperative actions to enhance the fitness of a close relative will be offset by the beneficial propagation of shared genetic material) [149,164] and reciprocal altruism [165] (the cost of an altruistic action is returned by a reciprocal action, either immediately or in the future, of the beneficiary) are the long standing explanations for the evolution of cooperation. It seems likely, given the constantly explorative nature of evolution, that cheaters would continually arise and test the fabric of cooperation. In fact, modeling suggests that cooperators and cheaters may stably coexist [166] and in some instances it is perceivable that cheating may be beneficial to a microbial ecosystem, as cheating may provide a means to reduce the pleiotropic nature of evolved complexity enabling the formation, or reconfiguration, of modules. Nevertheless, the evolution of cooperation requires evolutionary forces to assert limits on cheater tolerance. Potential mechanisms through which such limits are applied have been evaluated [167], though a firm theory remains to be adopted by the scientific community. For the microbiome, an additional checkpoint exists; MLS means selection also acts upon the fitness of the host and consequently the microbiome as a complete entity. The microbiome has clear routes to affect the fitness of the host (Figure 2) and consequently an irreparable perpetuation of cheaters amongst the microbiome would reduce the reproductive success of the host, which would in turn feedback to the evolutionary trajectory of the microbiome (Figure 2) [168].

## 6. Perspective

Despite several recent manuscripts incautiously prescribing the observed taxonomic differences entirely to a single factor such as diet [169], clear evidence exists to show each of the above-described evolutionary pressures play important roles in shaping the microbiome. These factors are not mutually exclusive; though breaking down their relative contributions is non-trivial. Only now, with the increasing number of animal microbiome data available, are we beginning to understand the nature of microbiome plasticity in animals [85,170]. With recent studies on the relative contributions of factors such as host genetics [128,170], diet [171], environmental exposure [87] and health [172], we are now poised to venture toward a quantified understanding of microbiome evolution. It is clear that a multifaceted approach is the only way we can hope to understand how a microbiome evolves and adapts along inter- and intra-specific axes, along with the varying contributions of polygenic traits on the abundance and interactions of individual microbial species, related taxa, and groups of distantly related organisms.
